# Crystal Structure, SAXS and Kinetic Mechanism of Hyperthermophilic ADP-Dependent Glucokinase from *Thermococcus litoralis* Reveal a Conserved Mechanism for Catalysis

**DOI:** 10.1371/journal.pone.0066687

**Published:** 2013-06-20

**Authors:** Jaime Andrés Rivas-Pardo, Alejandra Herrera-Morande, Victor Castro-Fernandez, Francisco J. Fernandez, M. Cristina Vega, Victoria Guixé

**Affiliations:** 1 Departamento de Biología, Facultad de Ciencias, Universidad de Chile, Santiago, Chile; 2 Centro de Investigaciones Biológicas (CIB-CSIC), Madrid, España; Institute of Enzymology of the Hungarian Academy of Science, Hungary

## Abstract

ADP-dependent glucokinases represent a unique family of kinases that belong to the ribokinase superfamily, being present mainly in hyperthermophilic archaea. For these enzymes there is no agreement about the magnitude of the structural transitions associated with ligand binding and whether they are meaningful to the function of the enzyme. We used the ADP-dependent glucokinase from *Termococcus litoralis* as a model to investigate the conformational changes observed in X-ray crystallographic structures upon substrate binding and to compare them with those determined in solution in order to understand their interplay with the glucokinase function. Initial velocity studies indicate that catalysis follows a sequential ordered mechanism that correlates with the structural transitions experienced by the enzyme in solution and in the crystal state. The combined data allowed us to resolve the open-closed conformational transition that accounts for the complete reaction cycle and to identify the corresponding clusters of aminoacids residues responsible for it. These results provide molecular bases for a general mechanism conserved across the ADP-dependent kinase family.

## Introduction

The Embden–Meyerhof pathway is the most common route for the degradation of glucose. Although this metabolic pathway is generally highly conserved between different organisms, several archaea of the *Euryarchaeota* have evolved major modifications with only four of the classical enzymes present in the canonical pathway. One of these major differences is the presence of ADP-dependent glucokinases (GKs) and phosphofructokinases (PFKs), instead of the classical ATP-dependent kinases [1], [2]. Although some authors have attributed the presence of ADP-dependent enzymes to a matter of metabolic adaptation to high temperatures and to starvation conditions, several facts indicate that the presence of these proteins in the central metabolism of archaea is not related to the hyperthermophilic life style [3]. Even though these ADP-dependent kinases show no sequence similarity to ATP-dependent enzymes known to date, the determination of their three dimensional structures allowed their reliable classification as members of the ribokinase superfamily. Structurally, these enzymes share a common Rossmann-like fold characterized by a α/β/α topology, which constitutes the large domain. In addition to this core ribokinase-like fold; other members of this superfamily have an extra small domain, which in the case of the ADP-dependent kinases is formed by a five stranded β-sheet with some α-helical insertions, the active site lying between the two domains. The small domain, which could function as an active-site lid to protect substrates from hydrolysis, has been proposed as a phylogenetic marker for the evolution of this superfamily [4].

To date the crystal structures of three ADP-dependent GKs are known [5]–[7]. These structures come from different organisms and were crystallized under experimental conditions that are not directly comparable. For example, Ito et al. [5] reported the first structure of an ADP-dependent kinase, the glucokinase from *T*. *litoralis* (TlGK) complexed with ADP. These authors found no evidence for a conformational change induced by ADP by comparing the ADP complex with the structure of the apo-TlGK, prepared by soaking a holo crystal in ADP-free solution, thereby raising the question as to whether an induced fit mechanism was required for catalysis. Later, Tsuge et al. [6] determined the crystal structure of apo-GK from *Pyrococcus horikoshii* (PhGK) at 2.0 Å resolution; when the structure of apo-PhGK was superimposed onto that of TlGK·ADP, a displacement by >5 Å of the small domain was observed, that led the authors to suggest that this large conformational change could take place during catalysis. Finally, the crystal structure of an ADP-GK from *Pyrococcus furiosus* (PfGK), complexed with ADPβS and glucose was reported, and the authors stressed the importance of glucose binding to achieve the closed conformation. They referred to the apo and ADP complexed forms as more open and open conformations [7].

Binding of ligands to proteins can trigger conformational changes, and conformational changes of proteins can increase binding affinity. The interplay between conformational changes and ligand binding is usually described in terms of induced fit or conformational selection models. The classical view corresponds to the work of Koshland and establishes an *induced fit* mechanism whereby ligand binding provokes a conformational change in the protein [8]. The other view, which has become more popular in recent years, suggests that the protein exists as an ensemble of conformations, a fraction of which is able to recognize and bind a particular ligand, favored by the intrinsic dynamics of the protein (*conformational selection*) [9], [10]. However, as reviewed by Vértessy and Orosz [11], this concept t was first described by the Hungarian biochemist F. B. Straub, who coined the term “fluctuation Fit” to hypothesize that proteins may exist in equilibrium between several different conformations. Also, other less used terms like “conformational selectivity”, “stabilization of conformational ensembles”, “population shift”, “selected fit” and “pre-existing equilibrium” have been used to describe this phenomenon. To date, many studies show an even more complex interplay between intrinsic dynamics and ligand-induced motions [12], [13]. Recently, Vogt and Di Cera have offered an explanation for the apparent infrequency reporting cases of conformational selection in the literature [14].

Since the crystal structures provide only static pictures defining snapshots along the catalytic cycle while necessarily missing about the dynamics and timing of the domain movements that occur in solution, it is necessary to assess whether the different conformations observed in the crystalline state are relevant to catalysis and related to the conformational behavior of the enzyme in solution. To address these questions for ADP-dependent TlGK, we employed a multidisciplinary approach combining the determination of the kinetic mechanism in order to evaluate the catalytically relevant enzyme forms to which substrate are bound, small angle X-ray scattering (SAXS) as a sensitive probe of the solution conformation in the absence and presence of ligands, and finally X-ray crystal structure determinations of the enzyme in the apo form and as a ternary complex form; this afforded the direct comparison of the structures of the same enzyme in all ligand states.

Our results provide insights into determinants of the structural changes that accompany ligand binding both in solution and in the crystalline state. We determine the binding order of each substrate to the active site in order to correlate the conformational changes that the enzyme experiences on the basis of its kinetic mechanism, with those observed by SAXS and X-ray crystallography. Detailed inspection of conserved clusters of residues involved in the open-closed conformational transition lead us to propose a general mechanism for the open-closed conformational transition that is a hallmark throughout the ADP-dependent kinases family.

## Results

### Kinetic Mechanism

**Figure 1:**
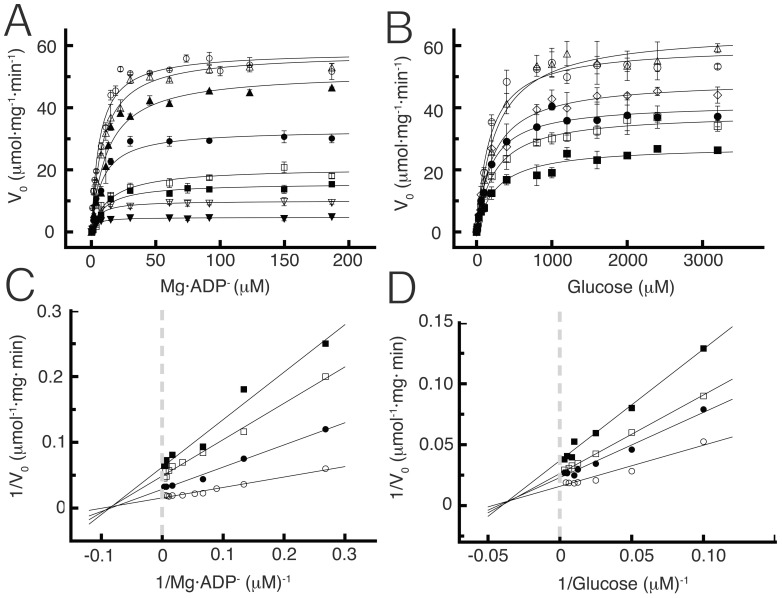
Initial velocity patterns for TlGK with D-glucose and MgADP as variable substrates. (**A**) Saturation curves for Mg·ADP at different constant concentrations of D-glucose. The fixed initial D-glucose concentrations were: 30 µM (▾), 50 µM (▿), 100 µM (▪), 150 µM (□), 200 µM (•), 250 µM (▴), 500 (▵) and 1000 µM (○). (**B**) Saturation curves for D-glucose at different constant MgADP concentrations; 10 µM (▪), 30 µM (□), 50 µM (•), 100 µM (◊), 300 µM (▵) and 1000 µM (○)MgADP. (**C**) Double reciprocal plots of curves shown in A. For clarity only four concentrations are depicted: 100, 150, 200 and 1000 µM D-glucose. (**D**) Double reciprocal plots of curves shown in B. For clarity only four concentrations are shown: 10, 30, 50 and 1000 µM Mg·ADP.

To elucidate the order of addition of substrates and release of products, steady-state kinetics approaches are followed to measure initial reaction rates and to test the nature of inhibition by product [15], [16]. In order to ascertain if the kinetic mechanism proceeds through a substituted-enzyme mechanism (ping-pong mechanism) or ternary complex formation (sequential mechanism), we performed initial velocity studies. TlGK presents a hyperbolic saturation curve for both substrates, Mg·ADP and D-glucose (Figure 1A and B). When Mg·ADP was varied at fixed D-glucose concentration an increase in the initial velocity was observed as D-glucose concentration was raised. The same trend was obtained when D-glucose was varied at fixed concentrations of Mg·ADP. In both cases, when data were analyzed using double reciprocal plots a family of lines intersecting above the abscissa was observed (Figure 1C and D). The observed intersection of lines is characteristic of a sequential mechanism where a ternary central complex must form before products are released. Table 1 summarizes the kinetic constants assuming a bi-substrate Bi-Bi model under steady state conditions, obtained from secondary plots of the slope and intercepts of double reciprocal plots.

**Table 1:** Kinetic parameters for ADP-dependent glucokinase from *T. litoralis*.

	K_M_ ‡(µM)	V_max_ ‡(µmol·mg^−1^·min^−1^)
MgADP	8.6	67.8
D-glucose	218.5	67.8

‡ Values obtained from fitting the data to the linear expression of bi-substrate ordered sequential model.

**Figure 2:**
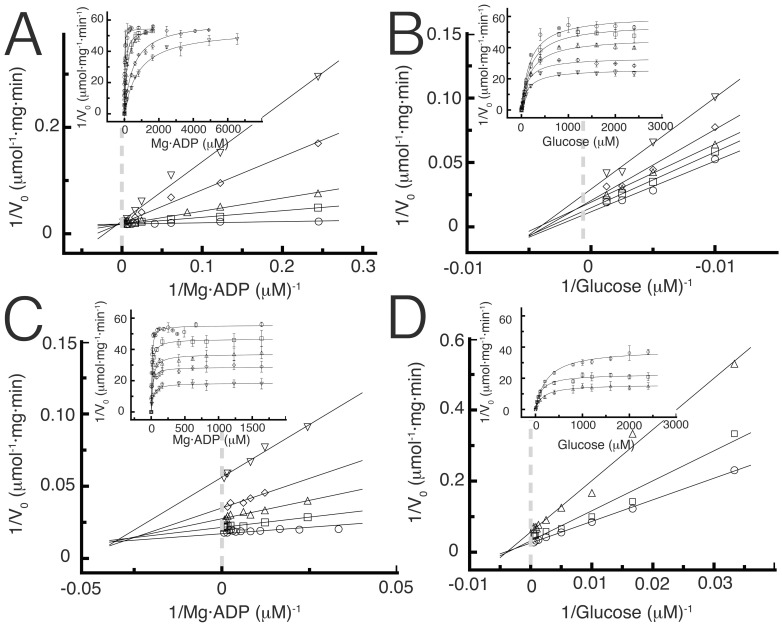
Product inhibition patterns for TlGK. (**A**) Inhibition by Mg·AMP with Mg·ADP as variable substrate. Measurements were assayed at fixed Mg·AMP concentrations: 100 µM (□), 200 µM (▵), 600 µM (◊) and 1000 µM (▿). Control curve in absence of product (○) was also included in the graph. (**B**) Inhibition by Mg·AMP with D-glucose as variable substrate. Measurements were assayed at fixed concentrations of Mg·AMP: 435 µM (□), 858 µM (▵), 1738 µM (◊) and 3657 µM (▿). Control curve in the absence of product (○) was also included in the graph. (**C**) Product inhibition by glucose-6-P with Mg·ADP as variable substrate. Measurements were assayed at fixed glucose-6-P concentrations: 100 µM (□), 500 µM (▵), 1000 µM (◊) and 2000 µM (▿). Control without the product was included (○). (**D**) Product inhibition by D-glucose-6-P with D-glucose as variable substrate. Measurements were assayed at fixed glucose-6-P concentrations: 500 µM (□) and 2000 µM (▵). Control curve without the presence of product was included (○). Inset of all figures show the non-linear fit of the total data.

**Figure 3:**
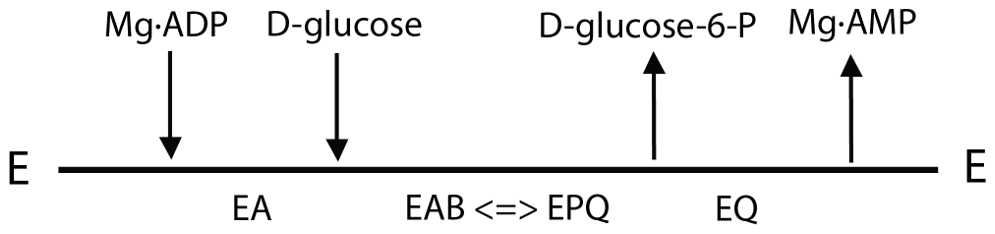
Sequential ordered Bi-Bi mechanisms for the reaction catalyzed by TlGK. Arrows indicate the entry and release of substrate and products.

To distinguish whether substrate binding to the active site was random or ordered, we performed product inhibition studies. Our results indicated that Mg·AMP behaves as a competitive inhibitor of Mg·ADP and as a mixed type inhibitor with respect to D-glucose; the other product, D-glucose-6-P presents a mixed type inhibition versus either Mg·ADP or D-glucose (Figure 2). These patterns of inhibition are consistent with an ordered sequential mechanism in which Mg·ADP is the first substrate to bind to the catalytic site and Mg·AMP the last product to be released. Replotting of the slopes and intercepts of the double reciprocal plots obtained by product inhibition showed a linear dependence of the slopes and intercepts with the inhibitor concentration (not shown) indicating the existence of a single product inhibition without the formation of dead end complexes. Our results fully agree with a reaction pathway involving the formation of a ternary complex as a compulsory step, which strongly support an ordered sequential. Table S1 summarizes the inhibition constants obtained from fitting the data to an ordered sequential mechanism, where substrate A is Mg·ADP and B is D-glucose (Figure 3).

### Ligand-induced Structural Transitions Observed by SAXS

In order to correlate catalytically relevant binding of substrates determined through the kinetic studies with the putative structural transitions induced by them, and to understand their interplay with the GK function we used solution small X-ray scattering (SAXS). This technique provides a sensitive probe of the macromolecular conformation in solution and has been applied to answer questions about conformation and conformational changes in several molecular systems [17].

**Figure 4:**
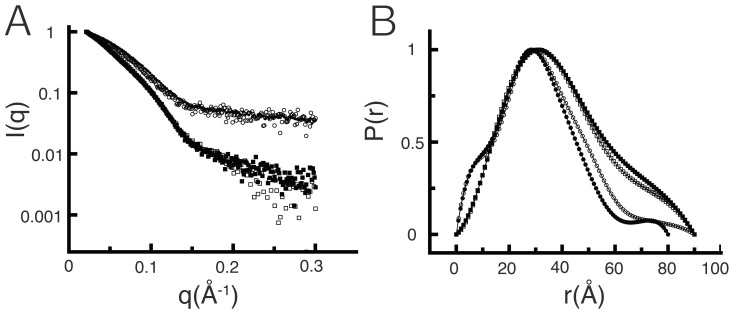
Scattering curves and pair distance distribution functions *P(r)*. (**A**) Scattering patterns for the different conditions explored: apoenzyme (□), enzyme-D-glucose (▪), enzyme-Mg·ADP (○) and in the presence of Mg·ADPβS and glucose (•). The curves were normalized to unity at their maximum value for comparison purposes. (**B**) P(r) graphs for each condition calculated by Fourier transformation using GNOM (28). The graphs were normalized to unity at their maximum value for comparison purposes.

Scattering curves for TlGK in solution demonstrated that the molecule changes its shape upon binding of the substrates, especially at angles q <0.15 Å^−1^. The shape of the scattering curves for the apo form as well as in the presence of D-glucose were almost identical, the only difference between them being a decrease in the dispersion of the data at angles q >0.2 Å^−1^ when D-glucose was present (Figure 4A). However, in the presence of Mg·ADP the scattering curve exhibited a dramatic change. The dispersion decreased and the slope of the scattering curve became clearly different, especially at angles q <0.1 Å^−1^ and at high angles q >0.2 Å^−1^ (Figure 4A). To obtain structural information of the ternary complex we used ADPβS as a nonhydrolyzable ADP analog, which binds to the active site but cannot participate as a substrate for the transfer reaction (data not shown). The scattering curve of the Mg·ADPβS·D-glucose complex, analogous to the ternary complex, had an even lower dispersion and the slope of the curve at high angles was similar to the one obtained for Mg·ADP, whereas at intermediate angles (q≈0.1 Å^−1^) a slope difference was observed (Figure 4A).

Analysis of the pair distance distribution functions *P(r)* revealed the conformational changes triggered by ligands. The radius of gyration (Rg) of the apo-enzyme was 29.0 Å while in the presence of Mg·ADP was reduced to 25.9 Å, confirming the formation of the E·A complex (Figure 4B). However, in the presence of D-glucose the measured Rg was 30.0 Å (Figure 4B), which is almost identical to the one obtained for the apo-enzyme (Table 2), indicating the absence of complex formation in the presence of glucose only. Other experimental approaches support the ligand binding events that were derived by kinetic and SAXS experiments. For example, isothermal titration calorimetry experiments demonstrated binding of MgADP to the enzyme in the absence of D-glucose, with dissociation constant close to the K_m_ value for this substrate (data not shown). Using the same strategy, we were unable to detect the formation of a TlGK·D-glucose complex. In the ternary complex, Rg further decreased to 24.0 Å (Table 2 and Figure 4B), indicating that the total conformational change was attained only after E·A·B complex formation. Similarly, fluorescent measurements following the Tryptophan signal from the TlGK shows an increase in intensity upon binding of glucose only in the presence of a ADP analog (AlF_3_-AMP) (data not shown). Thus, SAXS data suggest that changes in enzyme compactness recapitulated the sequential substrate binding events; binding of both substrates to the active site triggered a full domain closure involving a 5.0 Å reduction in Rg (Figure 4B and Table 2).

**Table 2:** TlGK radius of gyration (Rg) and distance between the small and large domains under different conditions.

	Apo-enzyme	D-Glucose	Mg·ADP	Mg·ADPβS·Glc
Rg^ξ^ (Å)	29.0±0.1	30.0±0.1	25.9±0.1	24.0±0.1
Rg^¶^ (Å)	24.1	N.A.	23.7	23.1
D^†^ (Å)	29.4	N.A.	28.1	26.5

Rg^ξ^ radius of gyration calculated using probability density function graphs. Rg^¶^ theoretical radius of gyration calculated with the software CRYSOL [48], using crystallographic data. D^†^ distance between the small and large domain calculate from crystal structures. For the apo-enzyme and the ternary ADPβS·D-glucose complex, we used the PDB structures solved in this work, whereas for the ADP complex the coordinates available from the PDB (1GC5) were used. N. A., not applicable.

**Figure 5:**
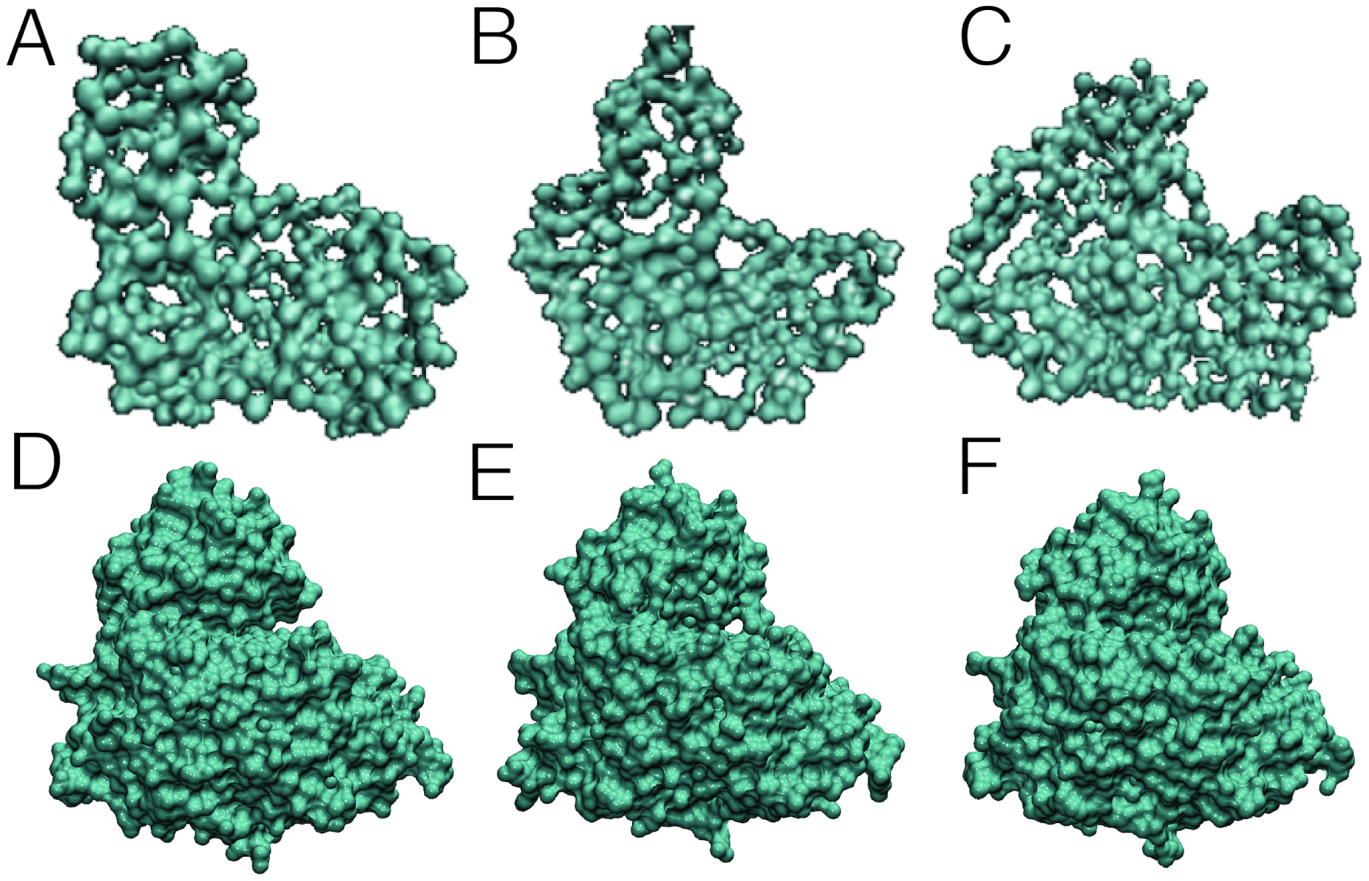
Comparison between SAXS envelope models and crystals structures. (**A**) Enzyme model obtained from the SAXS data in the absence of substrates; (**B**) in the presence of Mg·ADP and (**C**) in the presence of Mg·ADP and D-glucose. Every model was built using GASBOR with no symmetry constraints. (**D**), (**E**), (**F**) Surface representation of the enzyme’s structures in the absence of substrates; in the presence of ADP (PDB 1GC5) and in the presence of Mg·ADPβS and D-glucose, respectively.

*Ab initio* models built using the *P(r)* curves for each condition suggest that the relative position of the mass centers of the two domains changes as a function of the ligands added to the solution. For example, in the model built for the apo and the Mg·ADP conditions, the active-site cleft between the small and large domains is respectively widely or moderately open (Figure 5A and 5B), whereas in the presence of Mg·ADPβS·D-glucose, the small domain has moved toward the large domain to occupy a position that occludes the active site, suggesting that the enzyme has transitioned toward a fully closed state (Figure 5C).

### Structure Determination of the apo and holo forms of TlGK

To gain insight into the structural basis of TlGK catalysis, binding of substrates and associated conformational changes, we sought to obtain high-resolution crystal structures of this enzyme under different conditions. We determined the crystal structures of TlGK in its apo form (absence of ligands) and holo form, in the presence of D-glucose and the nonhydrolyzable ADP analog ADPβS (ternary complex, ADPβS·D-glucose). Both structures were obtained by molecular replacement, and refined to 2.05 and 2.58 Å resolution, respectively. X-ray data collection and refinement statistics for each structure are summarized in Table 3.

**Table 3:** Crystallographic data statistics and refinement.

	Apo-TlGK	Holo-TlGK(Mg·ADP*β*S·D-glucose)
**Data collection**		
Beamline	ID23-2	ID14-4
Wavelength (Å)	0.8726	0.9794
Space group	*P*3_2_21	*P*3_2_21
**Cell dimensions**		
*a*, *b*, *c* (Å)	109.1, 109.1, 129.6	106.5, 106.5, 130.1
*α, β, γ (*°)	90, 90, 120	90, 90, 120
Resolution (Å)	40.0–2.05 (2.10–2.05)*	35.0–2.58 (2.65–2.58)*
*R*_merge_	0.089 (0.564)	0.093 (0.672)
*I*/σ*I*	19.4 (3.7)	18.3 (3.5)
Total reflections	687,053 (72,183)	234,319 (33,911)
Unique reflections	56,090 (7710)	27,361 (1992)
Completeness	94.7 (99.2)	99.9 (100.0)
Redundancy	9.4 (12.2)	7.4 (7.4)
**Refinement**		
Resolution (Å)	39.3–2.05 (2.10–2.05)	34.9–2.58 (2.65–2.58)
No. reflections	55,935 (3529)	25,931 (1402)
*R*_work_/*R*_free_	0.17/0.21	0.17/0.22
**No. atoms**		
Proteins	3854	3810
ADP*β*S	–	23
D-glucose	–	13
Water	293	163
**B-factors (Å^2^)**		
Protein	34.6	50.5
AMP	–	50.2
D-glucose	–	49.9
Water	44.8	47.6
**R.m.s. deviations**		
Bond lengths (Å)	0.023	0.009
Bond angles (°)	2.058	1.360
**Ramachandran plot**		
Favored	457(98.3%)	450(97.0%)
Allowed	7(1.5%)	11(2.6%)
Outliers	1(0.2%)	2(0.4%)

**Figure 6:**
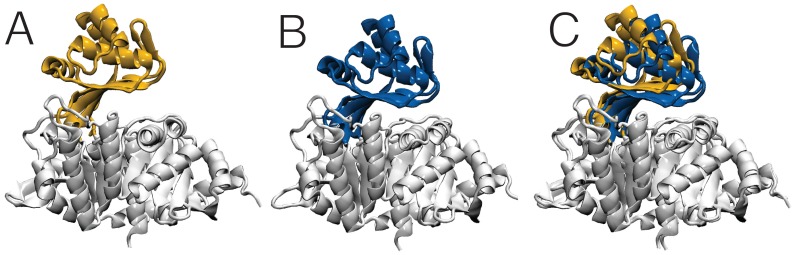
Structures of the *T. litoralis* glucokinase in the apo form and in the Mg·ADPβS·D-glucose ternary complex. (**A**) Ribbon representation of the enzyme in the absence of substrate. The large domain is shown in white, whereas the small domain is shown in yellow. (**B**) Ribbon representation of the enzyme in the presence of ADPβS and D-glucose. The large domain is shown in white and the small domain is shown in blue. (**C**) Structural alignment between the apo and holo (ADPβS·D-glucose) forms.

The overall structure of TlGK in both states (apo-enzyme and ternary complex) shows the same two domains organization described previously [5]; the large domain harbors a Rossmann fold (α/β/α) architecture with a central twelve stranded β-sheet surrounded by thirteen α-helices and three 3_10_ helices, and the small inserted domain consisting of five β-strands and four α-helices. The active site is located in a cleft between the two domains. The insertion of the small domain between strand 2 and helix 8 of the large domain at the farthest end of the active site cavity provides a physical basis for ligand-mediated communication between the two domains (Figure 6).

The spatial disposition of the two domains was different when comparing the structures of the apo-enzyme and the ternary complex (Figure 6). In the absence of ligands, the mass centers of the small and large domain were separated by 29.4 Å, but when Mg·ADPβS and D-glucose were simultaneously bound to the active site, the two domains underwent conformational changes causing the inter-domain distance to shrink by nearly 3 Å, that to 26.5 Å (Table 2). In the structure of TlGK·Mg·ADP reported previously [5], the distance between domains is 28.1 Å. Therefore, after binding of the first substrate (Mg·ADP) the distance between domains shrank by only 1.3 Å, whereas in the ternary complex, the inter-domain distance diminishes by an additional 1.6 Å, to complete a 2.9 Å closure movement (Figure 6C).

The conformational changes that occur upon sequential substrate binding can be explained by an almost pure rotation (or a rotation plus a translation) facilitated by residues in the flexible inter-domain connection. Structural changes that accompany binding of the first substrate (Mg·ADP) include a small relative rotation (4.8°) between the two domains plus additional movements that do not completely occlude the active site cavity. Accordingly, TlGK·Mg·ADP structure has been previously described as an “open” structure [5]. In contrast, the crystal structure of the ternary complex TlGK·Mg·ADPβS·D-glucose exhibits an 11.8° relative rotation with respect to the apo-TlGK structure and corresponds to a fully closed conformation that entraps both substrates in a Michaelis-Menten-like complex. This series of rotations recapitulate the sequential binding of substrates: binding of Mg·ADP leading to a 4.8° rotation, the second substrate causing a 7.2° rotation (TlGK·Mg·ADPβS·D-glucose versus TlGK·Mg·ADP). Thus, the total rotation to complete the active site closure is 11.7° (TlGK·Mg·ADPβS·D-glucose versus apo TlGK) (Figure S1). All these translational and rotational movements are accompanied by several charge distribution rearrangements at the inner space of the active site, which facilitate domain closure (Figure S2).

A full description of the interactions present at both binding sites (Mg·ADP and D-glucose) is illustrated in Supporting Information (Figure S3 and Figure S4).

**Figure 7:**
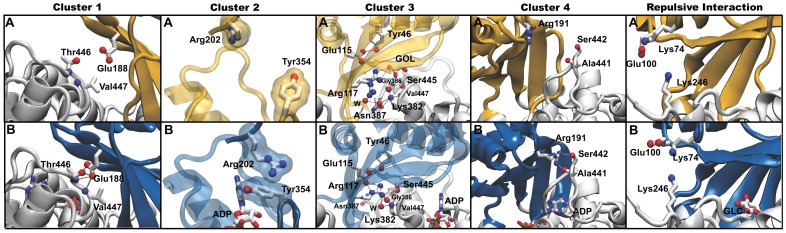
Interactions clusters between the small and large domain that mediate the transition from the open to the closed conformation. (A) Structure of the enzyme in the apo-form and (B) structure of the ternary TlGK·Mg·ADPβS·D-glucose complex. The small domain was colored in yellow (apo-enzyme) or blue (ternary complex) for clarity purposes. Side chains of residues are represented as sticks. The atoms involved in H-bonds are represented as spheres. The nucleotide (ADP), the cosolvent, glycerol (GOL) and water (W) are also shown. Cluster 1 achieves communication between the small and large domain through Glu188, Thr446 and Val447. Cluster 2 residues contribute to stabilize the ADP-induced conformational change. In the apo-enzyme, Arg202 of the large domain is over the active-site pocket and the side chain of Tyr354 is oriented toward the outside of the pocket. In contrast, in the TlGK·Mg·ADPβS·D-glucose ternary complex Arg202 and Tyr354 form a stacking interaction afforded by the rotation of their side-chains so as to allow a cation-π interaction. Cluster 3′s interaction network is rearranged upon ADP binding through the formation of H-bonds between the large and small domain that generates a net attraction. In cluster 4, Arg191 from the small domain H-bonds Ala441 and Ser442 from the small domain. The only net repulsive interaction is provided by cluster 5, involving Lys74 and Lys246; in the apo-enzyme Glu100 is H-bonded to Lys74 thus neutralizing its net charge, but in the ternary complex this stabilizing interaction is absent and Lys74 comes closer to Lys246 despite the associated unfavorable energy barrier.

Inspection of the TlGK·Mg·ADPβS·D-glucose structure reveals five clusters of interactions formed by residues from the large and the small domain that could mediate the transition from the open to the closed conformation (see below). Three of these clusters involve attractive interactions governed by hydrogen (H) bonds, a fourth one is an electrostatic attraction involving a cation-π interaction, and the fifth cluster, which consists of an intrinsically destabilizing interaction (an electrostatic repulsion), could play a regulatory role during domain opening by counteracting the net attractive interaction of the other four clusters (Figure 7).

Cluster 1 is formed by Glu188 (from the small domain) and residues Thr446 and Val447 (both from the large domain). In the open conformation, these residues are too far apart to interact, but when Mg·ADPβS·D-glucose is bound to the active site, the greater proximity between these residues afforded by the conformational change facilitates the formation of two H-bonds between the Glu188 side chain and the hydroxyl group of Thr446 and two other H-bonds with the backbone amide groups of Thr446 and Val447 (Figure 7, cluster 1).

Cluster 2 involves Arg202 (small domain) and Tyr354 (large domain). In the ternary complex, the guanidinium group of Arg202, relocates on top of the Tyr354 phenol side chain (large domain), establishing a new π-π cation interaction. The distance between the two side-chain groups is reduced from 12.0 Å in the apo-enzyme to only 3.4 Å in the ternary complex (Figure 7, cluster 2).

In Cluster 3, Arg117 from the small domain only can interact in the closed conformation with residues Gly386 and Ser445, both from the large domain. In the small domain, Tyr46 establishes an H bond with Glu115, which in turn accepts two H-bonds from Arg117. Therefore, the spatial location and polarity of Arg117 is highly influenced by the interactions that it established. On the other hand, Lys382 belonging to the large domain is located at 2.9 Å from Arg117 (in the ternary complex), forming three H-bonds with residues from the same domain: Gly386, Ser445, and Val447, which could influence the position and charge distribution of Lys382. Considering charge, distance and the microenvironment that surround Arg117 and Lys382 side chains, the net nature of this interaction is likely to be attractive (Figure 7, cluster 3).

Cluster 4 includes Ala441 and Ser442 from the large domain and Arg191 from the small domain. In the open conformation the side chain of Arg191 is too far away from Ala441 and Ser442 to interact, whereas in the closed conformation (ternary complex), Arg191 can donate two new H-bonds: one to the carbonyl group from Ala441 and another to the hydroxyl group from of Ser442 side chain (Figure 7, cluster 4).

Cluster 5 is defined by interactions between residues Lys74 and Lys246 located in the small and large domain, respectively. In the apo-enzyme the distance between amine groups is >8.7 Å, whereas in the ternary complex this distance decrease to 4.0 Å, close enough to establish a repulsive interaction between the side chains of these Lys residues. We hypothesize that this repulsive interaction that is formed only in the ternary complex could have a regulatory function by favoring domain opening as soon as the reaction products abandon the active site (Figure 7, repulsive interaction).

### Conserved Interactions Involved in the Open-closed Transition in the ADP-dependent Kinase Family

**Figure 8:**
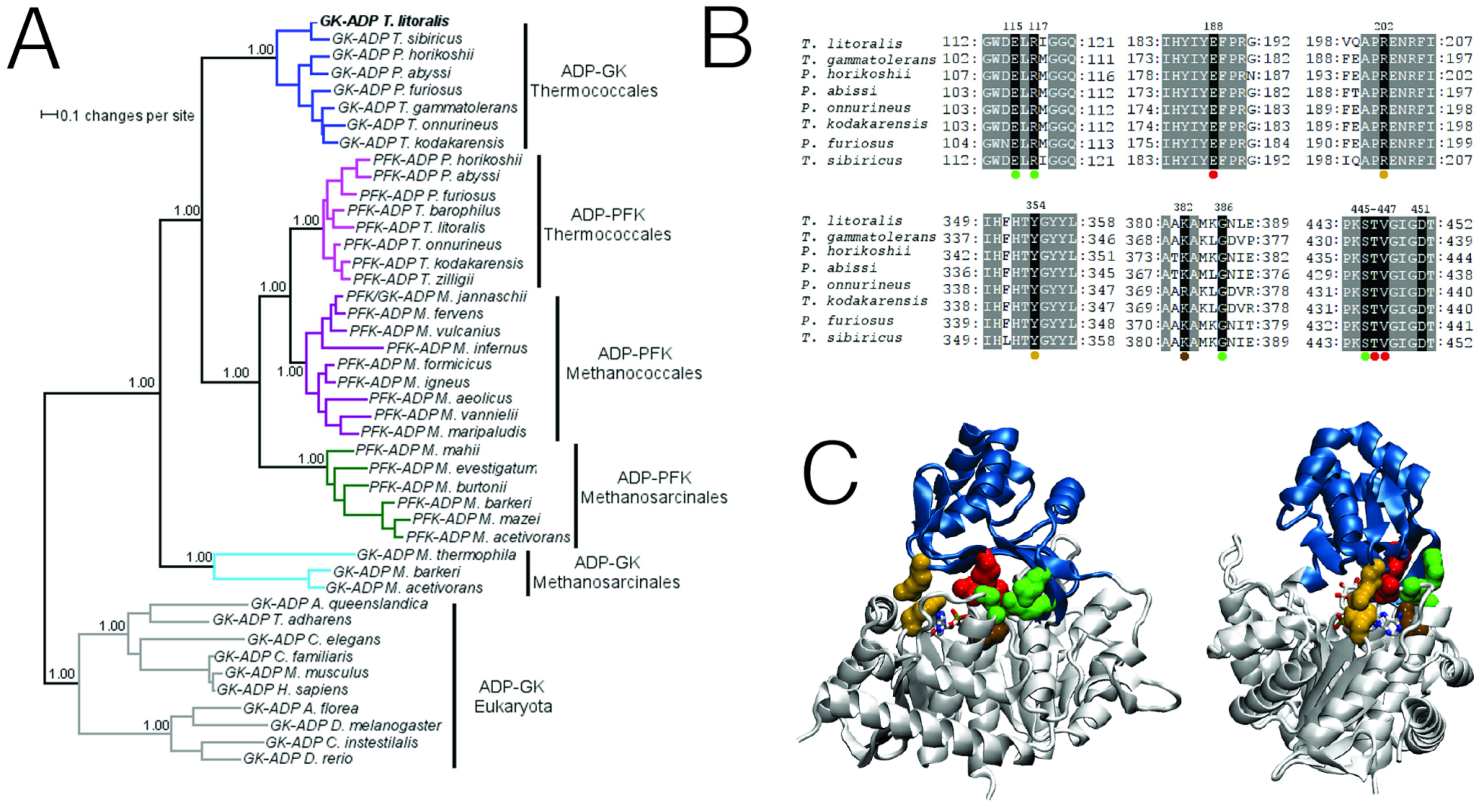
Conservation of cluster residues involved in domain opening/closure across the ADP-dependent sugar kinases family. (**A**) Consensus phylogenetic tree for ADP-dependent sugar kinase family determined by Bayesian inference. Groups of enzymes from archaea are shown in color: GK from *Thermococcales* (blue), PFK from *Thermococcales* (pink), PFK from *Methanococcales* (purple) PFK from *Methanosarcinales* (dark green) and GK from *Methanosarcinales* (cyan). The group of enzymes from eukaryotic organisms that were used as an outgroup to establish the tree root is shown in gray. The posterior probability of some interesting groups is shown in its respective node. (**B**) Multiple sequence alignment of glucokinases from the *Thermococcales* group. Residues involved in clusters described in the texts are indicated by dots; cluster 1, red (Glu188-Thr446/Val447); cluster 2, yellow (Arg202-Tyr354); cluster 3, green (Arg117/Glu115-Gly386/Ser445) and brown (Lys382-Arg117). (**C**) Amino acids involved in clusters 1–3 are shown as spheres and colored as in B.

To evaluate whether the existence of clusters of residues involved in attractive and repulsive interactions between both domains corresponds to a conserved strategy among the ADP-dependent sugar kinase family, we analyzed the evolutionary profile of this family obtained by Bayesian inference of phylogeny the interacting residues are conserved. In the profile obtained (Figure 8A) all the groups represented in the tree correspond to the following archaeal orders: ADP-GKs from *Thermococcales* (blue), ADP-PFKs from *Thermococcales* (pink), ADP-PFKs from *Methanococcales* (purple), ADP-PFKs from *Methanosarcinales* (green), ADP-GKs from *Methanosarcinales* (cyan); except ADP-GK from Eukarya (gray) which was used as outgroup. Analysis of both the sequences employed to construct the tree and the equivalent structural positions of the interactions involved in the opening/closing domain revealed that some interactions are highly conserved (Figure 8B and 8C). For example, in cluster 1 residues Glu188, Thr446 and Val447 are fully conserved across the whole family. On the other hand, residues involved in cation-π interactions (cluster 2) are conserved in all ADP-dependent glucokinases from hyperthermophilic organisms belonging to the *Thermococcales* group. Residues from cluster 3, which participate in the attractive interactions between Arg117 and Ser445/Gly386, are conserved in all GKs and PFKs from thermophilic archaea (*Thermococcales* and *Methanococcales*). Interestingly, in the context of their repulsive interaction, Arg117 and Lys382 are conserved in all the enzymes of the thermophilic organisms analyzed in our study. Residues of cluster 4 and 5 are only present in some sequences and therefore are less well conserved.

## Discussion

Understanding the mechanism of molecular recognition and in particular the characteristics of the response of a macromolecule to binding of a small molecule binding represents a challenging issue of modern structural biology. Our studies concerning the kinetic properties of ADP-GK in solution, along with the SAXS and X-ray crystallographic data provide important new perspectives on the conformational changes and dynamics of TlGK during catalysis, which are also relevant for other ADP-dependent hyperthermophilic kinases. The results support the idea that the enzyme is conformationally flexible and sensitive to specific substrate binding events. When the enzyme is in ligand-free state (apo), the large and small domains are distant from each other and accordingly there are no discernible interactions between residues from either domain. In contrast, for the ternary TlGK·Mg·ADP·D-glucose complex to become properly assembled, the two domains of the holo enzyme are rearranged by a twisting and closing motion that leads to a fully closed, catalytically competent conformation.

Comparison of the molecular envelope calculated from the SAXS data with those obtained from the crystal structures allows us to identify differences between the relative positions of the small and large domains in different ligation states (Figure 5). For the apo-enzyme, the SAXS *ab initio* model suggests a more open conformation than the one found in the crystal. Differences are also found when comparing the ternary complex condition; in this case the SAXS envelope suggests a more closed conformation than the one observed in the crystalline structure. Thus, although the crystal structure indicates that the enzyme undergoes domain closure (Figure 6C), the SAXS results suggest that this conformational change is even more dramatic in solution; the apo form is more open and the holo form is more closed than the ones obtained by X-ray diffraction (Figure 5A, 5C and Table 2). In fact, the difference between the theoretical Rg calculated for the crystal structures of the apo and holo enzymes is only 1 Å, whereas the Rg calculated from the *P(r)* indicate a domain closure of 5 Å (Table 2). This situation could reflect the larger conformational freedom of the enzyme in solution compared to the crystalline state. In spite of this, the most striking structural aspect about nucleotide and D-glucose binding to the active site is that both binding events indicate a sequential conformational change, in complete agreement with the orderly entry of substrates determined from the kinetic mechanism. Mg·ADP triggers the first structural change (semi-closed conformation), which favors the oncoming of the small domain toward the large domain, followed by the entry of D-glucose, which in turn leads to ternary complex formation and total domain closure. This sequential conformational change is a strong suggestion for an induced-fit mechanism [8]. In our case, movement toward the catalytically active conformation results in the occlusion of the active site. It has been proposed that this mechanism would favor catalysis by precluding solvent from competing with the catalyzed reaction but domain closure could also impair catalysis by restricting binding of the second substrate to this enzyme form. The latter possibility raises the question as to whether such a situation is even compatible with a conformational selection model since, even if the enzyme samples this semi-closed state in solution, steric occlusion of the active-site cleft might impede substrate binding. Moreover, Sullivan and Holyoak [18] have proposed that enzymes with lid-gated active sites must operate by an induced fit mechanism instead of conformational selection, and Okazaki and Takada [12] reported that stronger and long-range interactions favor induced fit, whereas shorter-range interactions favor conformational selection. However, although the preceding evidence supports an induced fit mechanism, it has been stressed that both mechanisms are likely to play important roles in molecular recognition and that these models are not mutually exclusive.

For example, even if initial binding were achieved by a conformational selection mechanism, it is likely that further changes in protein structure and energy landscape must occur in order to achieve optimal intermolecular interactions, which undoubtedly constitutes an induced fit process. The co-existence of both mechanisms is reported to exist in maltose binding protein [19] whereas the induced fit mechanism has been proposed for other mesophilic enzymes belonging to the superfamily, such as ribokinase and adenosine kinase [20]–[22]. In the case of ribokinase, it has been suggested that domain closing is a prerequisite for nucleotide binding [20] whilst Ito *et al* [6] propose that access to the nucleotide binding site of TlGK is more difficult after domain closing and propose that ADP binds weakly to the open conformation with little or absent conformational change, as opposed to what was described in the present study.

Another important point to be considered is how the enzyme coordinates its structural elements to assume the closed conformation and how, once this conformation is stabilized. The interaction between the large and small domain (via specific residue clusters), could represent a successfully strategy to achieved this goal. All the clusters described communicate both domains by non-covalent bonds and some of them are conserved in other enzyme homologs. Conservation analysis of cluster 1 shows that Glu188, from the small domain, and Thr446 and Val447, both in the large domain, are conserved across the whole family. This feature allows us to infer that net attractive interaction network between the GK large and small domains has been conserved during evolution in thermophilic and mesophilic enzymes of archaea as well as in the eukaryote domain, thereby implying that this interaction must already be present in the last common ancestor of the whole family.

On the other hand, the cation-π interaction between the side chain of Arg202 and Tyr354 (cluster 2) is a very sophisticated strategy dedicated to stabilize the closed conformation (Figure 7). Noticeably, as in cluster 1, these interactions are not exclusive of the TlGK enzyme, rather they are a common feature present in all the hyperthermophilic ADP-dependent GKs from the *Thermococcales* group while absent in mesophilic glucokinases (Figure 8). This class of interaction was studied by Minoux and Chipot [23], using a molecular dynamics approach in 1718 proteins. Their results show that π-system interactions (cation-π and π-stacking interaction) are usually established between arginine and tyrosine residues. Moreover, the distances between the interacting π-systems calculated *in silico* by these authors (3.6 Å) are very similar to the ones seen in the crystal structure of the ternary complex of the TlGK (3.4Å) (Figure 7 cluster 2). In another study, Gromiha *et al*. [24] suggested that in thermophilic proteins the π-system interaction frequently employs Tyr residues, instead of other aromatic residues such as Phe or Trp.

In the last few years there is an increasing number of reports that highlight the relevance of cation-π interactions in biological systems. These interactions have been demonstrated to have a substantial impact on protein structure as well as in catalysis and organic synthesis. For example, using a multidisciplinary approach Pecsi et al [25] showed that elimination of the aromatic interaction had a little effect on substrate binding but a major impact in the catalytic efficiency of dUTPase. Based on the similar position of these aromatic interactions in various nucleotide-hydrolyzing enzymes, the authors proposed that this kind of interactions could be a general component of the enzymatic catalysis of phosphate ester hydrolysis. The biological relevance of cation-π interactions are illustrated, among others examples, by the critical role that they play in nicotine addiction, their prevalence at protein-protein interfaces, and their contribution to the understanding of thermo or psychro stabilizing interactions [26], [27].

In the case of the cluster 3, residues involved in the attractive interaction between Arg117 from the small domain and residues Ser445 and Gly386 from the large domain (Figure 7) are conserved in all the thermophilic PFKs and GKs, but not in the mesophilic enzymes. The same scenario is seen with the repulsive interaction present in the same cluster, formed by residues Arg117 and Lys382, where the interaction is conserved in all thermophilic proteins of the family but not in the mesophillic members.

Finally, cluster 5 involves repulsive interactions between two lysine residues; none of them are conserved in any homolog considered in our analysis. In particular, this cluster seems to be exclusive of TlGK. Theoretical prediction of the protonation state of these lysine residues, using the PDB2PQR server implemented for electrostatics calculations [28], indicate that both residues should be positively charged at the pH employed for catalysis and therefore repel one another, facilitating domain opening. Interactions of this nature were deeply studied by Vondrášek *et al*. [29] who, using an *in silico* approach, analyzed the lysine-lysine and arginine-arginine ion pair. The authors compared their results with analysis of structural databases, indicating that there is a strong repulsive effect between the ammonium groups (NH_4_^+^) of lysine. This effect is due to charged ammonium ion and its almost spherical geometry, which favors repulsive Coulomb potential.

In summary, the closed conformation could be stabilized by at least four sets of attractive interactions between the small and large domain: one cation-π (cluster 2) and three H-bonds clusters (clusters1, 3 and 4), which in total form 7 formally attractive interactions (considering only two H-bonds for Glu188). Cluster 5 contributes one repulsive interaction between the large and small domain and could therefore facilitate domain opening and product release. In total, 6 H-bonds and 1charge-charge interaction support domain closing whereas 1 electrostatic repulsive interaction favors the opening. Preliminary molecular dynamics studies used to evaluate the behavior of each cluster commented above, confirmed the attractive nature for the cluster 1, 2 and 4 and the repulsive character of clusters 3 and 5.

A similar scenario has been described for phosphoglycerate kinase [30]. This enzyme would works as a spring, whereby the entry of the substrate favors domain closing which, in turn, results in the exposure of a hydrophobic patch that quickly triggers domain opening. Also, an H-bond network that stabilizes domain closure has been described for this enzyme [31].

Here we present an integrated approach that determines the conformational changes experienced by a hyperthermophilic enzyme (TlGK) in solution and in the crystalline state and their correlation with the kinetic mechanism. The results support an induced-fit mechanism although the participation of a conformational selection mechanism, at least in some reaction steps, cannot be ruled out. Also, we demonstrate the presence of conserved cluster of residues that could be involved in the stabilization of the closed conformation, as well as another one that could account for domain opening. Some of these interactions are fully conserved in the whole family of ADP-dependent kinases, hence constituting the basis for a general mechanism involved in catalysis, which may prove general for many other hyperthermophilic enzymes.

## Materials and Methods

### Purification of TlGK

The enzyme was purified as described by Merino *et al*, [32]. Briefly, the cells from 1L of LB broth were harvested by centrifugation and disrupted by sonication. Enzyme purification consisted basically in a thermal shock followed by (NH4)_2_SO_4_ precipitation and two-chromatographic steps (hydrophobic and ionic exchange chromatography). Every step during purification was performed in 50 mM Tris-HCl pH 7.8, and 5 mM MgCl_2_. Prior to any kinetic or SAXS measurement, the buffer was exchanged to 50 mM Hepes-NaOH pH 7.8 and 5 mM MgCl_2_.

### Kinetic Experiments

Enzyme activity was measured spectrophotometrically by following NAD^+^ reduction at 340 nm coupled with D-glucose-6-phosphate oxidation at 40°C. Standard assay was carried out in a final volume of 0.7 mL in the presence of 25 mM Hepes pH 7.8, 0.5 mM NAD^+^, 2–5 U of D-glucose-6-P dehydrogenase and the indicated concentrations of substrates (ADP and glucose). In all initial velocity studies performed, the concentration of free Mg^2+^ was kept constant.

The same spectrophotometric method was used to determine product inhibition kinetics with Mg·AMP, whereas for glucose-6-P a discontinuous assay was employed whereby the disappearance of Mg·ADP is quantified by the formation of pyruvate and its subsequent oxidation to lactate. The reaction mixture for the discontinuous assay contains 0.2 mM NADH, 100 mM KCl, 4 U of pyruvate kinase and 4 U of lactate dehydrogenase, in 1 mL of reaction.

### Kinetic Data Analysis

The data from initial velocity experiments were analyzed using non-linear hyperbolic plot and double reciprocal plots, both considering a kinetic model of a Bi-Bi ordered sequential mechanism (Equation 1). The kinetics constants were obtained from secondary plots of the slopes and intercepts of double reciprocal plots. For product inhibition experiments, the inhibition pattern and inhibition constant were analyzed by plotting the slopes and intercepts of the primary double reciprocal plots against product concentration. The Marquardt-Levenberg algorithm was used for the non-linear and linear adjustment of the kinetic parameters. The weighting system used for our fit was r2, giving equal weight to each value included in the kinetic measurements. Kinetic constants calculated by global fit using the program Visual Enzymics (SoftZymics) (2010) and by linear regression of the reciprocal plots, were essentially the same.

The nomenclature used in the initial velocity expression is according to Cleland [33].

Equation 1
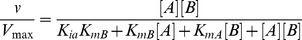



### SAXS Measurements and Data Processing

All scattering data were collected in the beamline D11A-SAXS1 [34], in the National Synchrotron Light Laboratory (Campinas, SP, Brazil). A wavelength λ 1.488 Å and a sample-to-detector distance of 1000 mm were used. The scattering data were recorded using the photon-counting Pilatus detector. The magnitude of the scattering vector, defined as q = 4πsinθ/λ (where 2θ is the scattering angle) was ranged 0.001 nm ^−1^< q <0.3 Å^−1^. For every experimental condition the temperature was maintained at 40°C and samples were irradiated for 3 or 5 min, collecting several spectra to monitor radiation damage and beam stability. The buffer scattering was subtracted for each condition and the resulting curve was analyzed. For each condition employed, several protein concentrations were assayed in order to ascertain the influence of protein aggregation in the scattering of the sample. TlGK at 1–6 mg·mL^−1^ were used, observing significant changes at low angle only when protein concentration exceeded 5 mg·mL^−1^; hence further analyses were restricted to the scattering data from protein samples at 4 mg·mL^−1^. Radii of gyration were measured using the Guinier approximation considering angles where q <1.3/Rg. We also evaluated the Rg by the pair distance distribution function *P(r)* calculated with the software GNOM [35]. *Ab initio* models of the enzyme were built using the software GASBOR [36].

### Crystallization, Data Collection and Structure Solution

TlGK was concentrated to 15 mg·mL^−1^ in storage buffer. Sitting-drop vapor-diffusion crystallization experiments were set up by mixing 1 µL protein solution and 1 µL crystallization condition over 75 µL mother liquor. Crystals of apo-TlGK were obtained in 14% (w/v) polyethylene glycol (PEG) 6000, 0.2 M LiSO_4_, 0.1 M sodium citrate pH 3.6 and 5 mM dithiothreitol (DTT). Crystals of TlGK·Mg·ADPβS·D-glucose were obtained in 1.5 M sodium citrate pH 5.2, 5 mM DTT, 5 mM MgCl_2,_ 6 mM ADPβS and 30 mM D-glucose. Both apo-TlGK and TlGK·Mg·ADPβS·D-glucose- crystals were cryo-protected by direct addition of 20% (v/v) sterile glycerol and flash-frozen in liquid nitrogen.

Complete and redundant X-ray diffraction data sets were collected at the ID23-2 (apo-TlGK, to a resolution of 2.05 Å) and ID14-4 (TlGK·Mg·ADPβS·D-glucose-, to a resolution of 2.58 Å) beamlines at the European Synchrotron Radiation Facility (ESRF, Grenoble, France). Data were indexed and integrated with XDS [37] and scaled and merged with SCALA [38] from the CCP4 software package [39]. Crystallographic data processing and merging statistics are summarized in Table 3. The structures of TlGK·Mg·ADPβS·D-glucose and apo-TlGK were phased by molecular replacement with PHASER 2.5 [40] using the semi-open structure of TlGK·Mg·ADP determined previously (PDB 1GC5) [5]. Minimally biased maps of the molecular replacement solutions calculated before refinement showed conspicuous density differences between the search model and the structure of TlGK·Mg·ADPβS·D-glucose· consistent with the presence of bound substrates and accompanied by a significant inter-domain movement. ADPβS was modeled as AMP since electron density for the β-phosphate was missing in the electron density maps. The structures were iteratively built using Coot [41] and refined with REFMAC5 [42]. Upon convergence, the R_work_/R_free_ for the apo-TlGK and TlGK·Mg·ADPβS·D-glucose structures were 0.17/0.21 and 0.17/0.22, respectively, and both had good geometry and stereochemistry as assessed by MolProbity [43]. Refinement and validation statistics are summarized in Table 3. All figures of protein structures were prepared with VMD [44].

### Accession Numbers

The X-ray crystal structures have been deposited with the Protein Data Bank with accession codes 4B8R (apo-TlGK) and 4B8S (TlGK·Mg·ADPβS·D-glucose).

### Alignment and Bayesian Inference of Phylogeny

The protein sequences were extracted using the protein BLAST Server from non-redundant protein sequence database (nr) using the PSI-BLAST algorithm with 5 iterations, using ADP-GK from *T. litoralis*, ADP-PFK from *P. horokoshii* and ADP-GK from *H. sapiens* as templates. The multiple sequence alignment (MSA) was constructed based on three-dimensional and secondary structure constraints using Promals3D [45], and then misaligned positions were corrected manually. To build an evolutionary profile of the family, redundant sequences in eukaryotes were removed using QR Sequence tool of Multiseq in VMD [46] with a PID 40%. Finally gaps positions were removed using Seqverter and the MSA was prepared for Bayesian inference of phylogeny with Mr. Bayes version 3.1.2 [47]. For the analysis we used Cprev as the fixed rate model, which showed a posterior probability of 1.0 in the mixed model and gamma-shaped rate variation across site with a proportion of invariable site. The number of generations was set to 1×10^6^, samplefreq to 100, with 2 run with 4 chains per run and temperature parameter set to 0.2. The average standard deviation of the split frequencies was less than 0.01 upon convergence. The consensus tree was calculated from 15,002 samples trees.

## Supporting Information

Figure S1 **Structural alignment of TlGK structures.**
(DOCX)

Figure S2 **Representation of Molecular Electrostatic Potentials.**
(DOCX)

Figure S3 **TlGK substrate contacts.**
(DOCX)

Figure S4 **Nucleotide and sugar binding sites.**
(DOCX)

Table S1 **Product inhibition parameters.**
(DOCX)
