# Supplementary material for: Crystal Structure, SAXS and Kinetic Mechanism of Hyperthermophilic ADP-Dependent Glucokinase from Thermococcus litoralis Reveal a Conserved Mechanism for Catalysis
Source: PLoS One. 2013 Jun 20;8(6):e66687. doi: 10.1371/journal.pone.0066687 (PMC3688580; doi:10.1371/journal.pone.0066687)
Supplement: Figure S1 — Structural alignment of TlGK structures. (DOCX) [file pone.0066687.s001.docx]

**
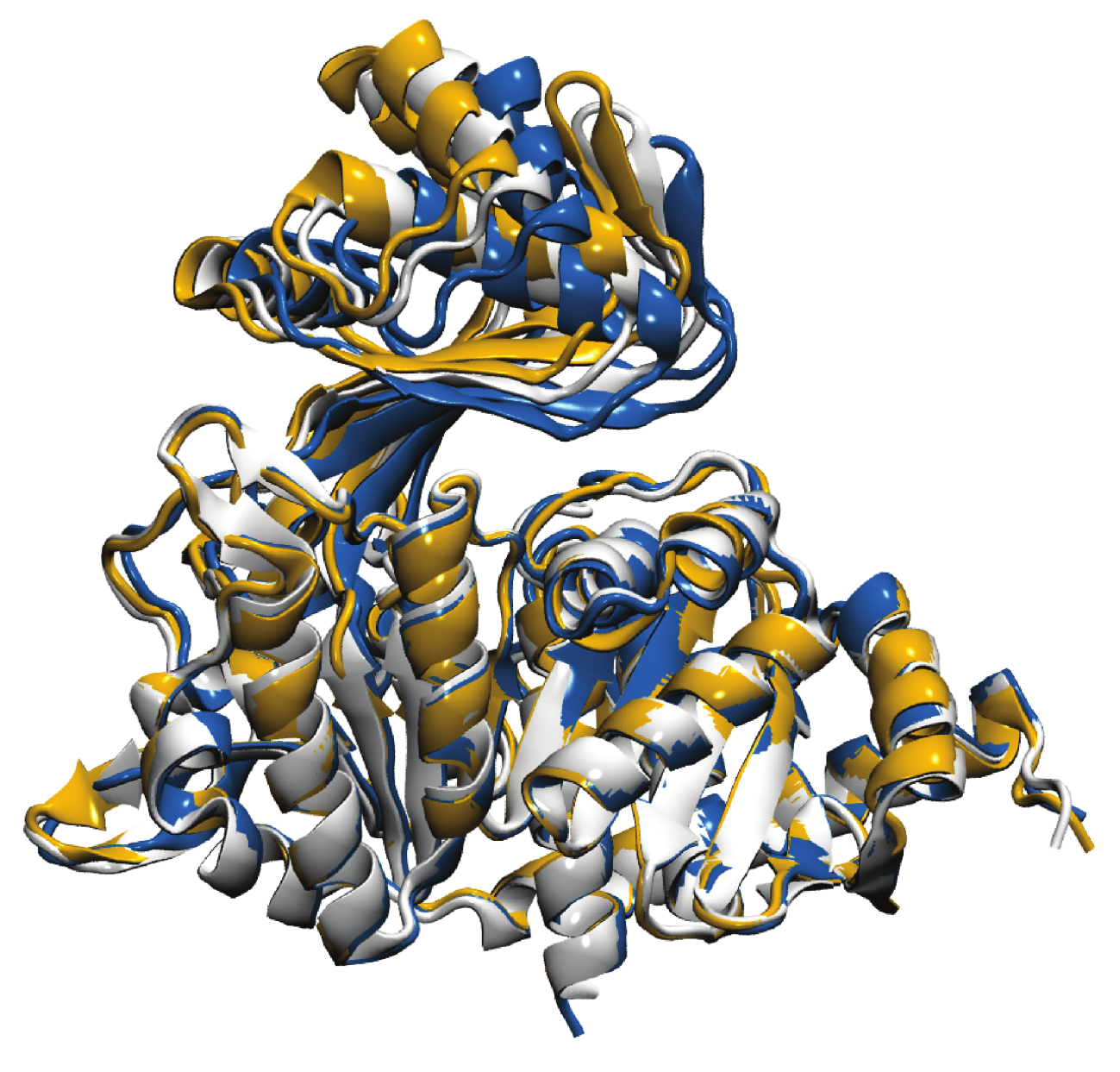
**

**Figure S1. Structural alignment of TlGK structures.** Apo TlGK (yellow), TlGK·Mg·ADP described by Ito et al. 2001 (grey) and TlGK·Mg·ADPβS·D-glucose (blue).

Ito S, Fushinobu S, Yoshioka I, Koga S, Matsuzawa H, Wakagi T (2001) Structural basis for the ADP-specificity of a novel glucokinase from a hyperthermophilic archaeon. Structure 9: 205-214.
