# Supplementary material for: Crystal Structure, SAXS and Kinetic Mechanism of Hyperthermophilic ADP-Dependent Glucokinase from Thermococcus litoralis Reveal a Conserved Mechanism for Catalysis
Source: PLoS One. 2013 Jun 20;8(6):e66687. doi: 10.1371/journal.pone.0066687 (PMC3688580; doi:10.1371/journal.pone.0066687)
Supplement: Figure S2 — Representation of Molecular Electrostatic Potentials. (DOCX) [file pone.0066687.s002.docx]

**Electrostatic Analysis of the enzyme TlGK**

Electrostatic analysis using the APBS tools available in PyMOL (DeLano, WL 2002) of the crystal structures shows a large rearrangement of the charge distribution at the inner space of the active site, especially after ADP binding (Figure S1A and C). The electrostatic map in the apo-enzyme shows a relatively neutral character that is altered after nucleotide binding, eliciting a change to a positive character. In the structure with ADPβS·D-glucose the active site is totally occluded by the small domain and the solvent accessible surface area leaves only a small space between both domains, thereby preventing access or release of further molecules (Figure S1A versus E, and B versus F).


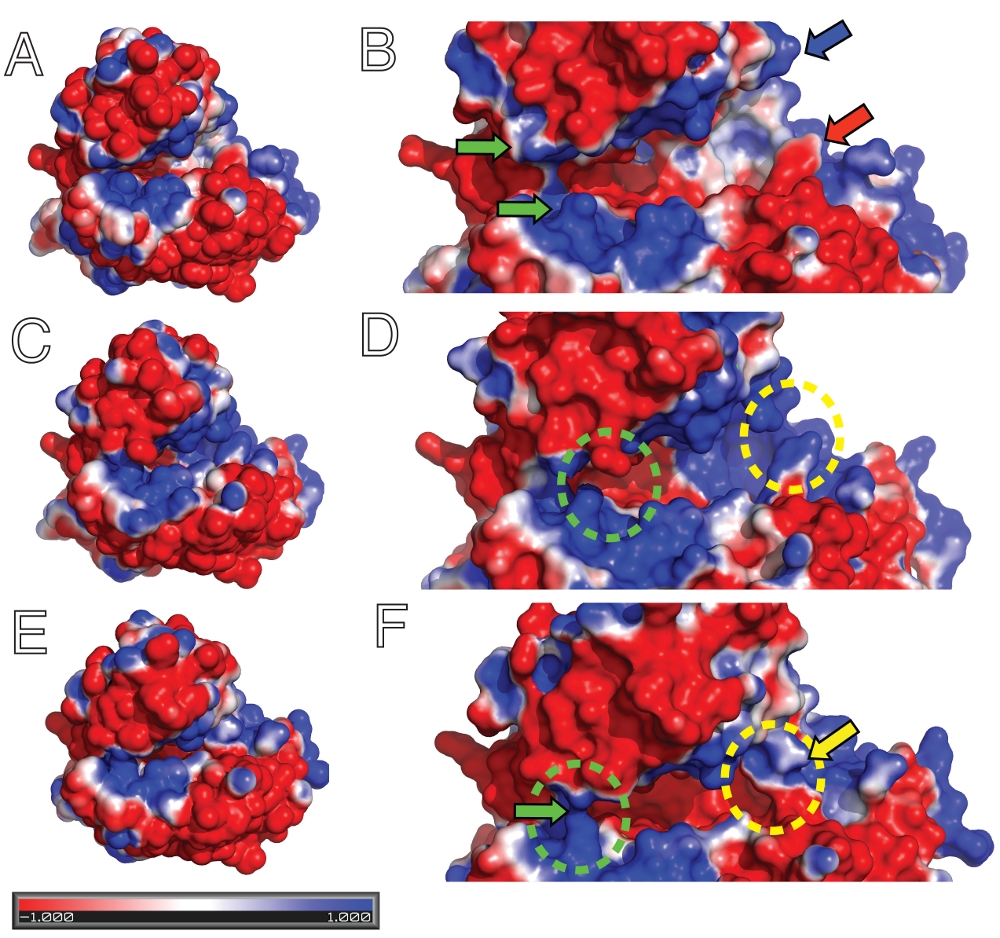


**Figure S2. Representation of Molecular Electrostatic Potentials distribution of TlGK in the open to closed transition.** (A) (C) and (E) Electrostatic potential representation of the solvent accessible surface in the apo-enzyme, TlGK·Mg·ADP and TlGK·Mg·ADPβS·D-glucose, respectively. (B) (D) and (F) Surface representation of the enzyme using the same electrostatic map, under different conditions: apo-enzyme, TlGK·Mg·ADP and TlGK·Mg·ADPβS·D-glc, respectively. Bar scale ranges from +1 Kcal/mol to -1Kcal/mol, using the blue color for positive charge, white for neutral, and red for negative charge. The arrows indicate the position of the Arg202 (blue arrow), Tyr354 (red arrow) and Lys74 and Lys246 (green arrows).

DeLano, WL (2002) The PyMOL Molecular Graphics System, DeLano Scientific LLC, San Carlos, CA.
