# Supplementary material for: Crystal Structure, SAXS and Kinetic Mechanism of Hyperthermophilic ADP-Dependent Glucokinase from Thermococcus litoralis Reveal a Conserved Mechanism for Catalysis
Source: PLoS One. 2013 Jun 20;8(6):e66687. doi: 10.1371/journal.pone.0066687 (PMC3688580; doi:10.1371/journal.pone.0066687)
Supplement: Figure S3 — TlGK substrate contacts. (DOCX) [file pone.0066687.s003.docx]

**
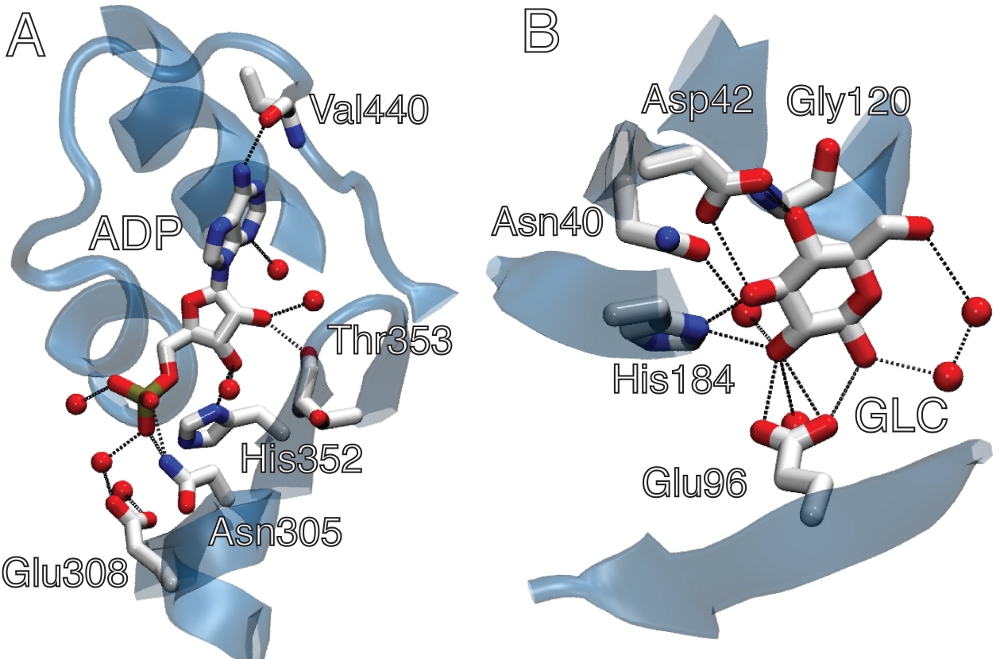
**

**Figure S3. TlGK substrate contacts.** **(A)** View of the ADP binding pocket in the TlGK ADPβS·D-glucose ternary complex. Val440 established H bonds with the NH^6^ of the ADP, while residues Thr353 and His352 established this kind of interaction with positions 2’ and 3’ of the ribose moiety, respectively. Asn305 makes contact with the oxygen from the α-phosphate through two H-bonds. Five water molecules establish H bonds with the nucleotide, one in the N^3^ from the adenine moiety, two with the ribose in the position 2’ and 3’ and two more with the oxygen from the α-phosphate. In the second sphere, Glu308 of the conserved NXXE motif accepts H bonds from water molecules (shown as red spheres). **(B)** View of the glucose binding pocket in the TlGK ADPβS·D-glucose complex. Asp42 establish two H-bonds with the 3’ and 4’ of the glucose, Glu96 can accept two H bonds from positions 1’ and 2’ of D-glucose, His184 is hydrogen bonded to to positions 2’ and 3’ of glucose. Finally, the amine group from Gly120 is involved in an H bond with position 4’ of the D-glucose. Furthermore, four water molecules interact with the sugar molecule in positions 1’, 2’ and 6’. Hydrogen bonds are represented by dashed lines. Side chains and adenosine and glucose atoms are in blue for nitrogen atoms, red for oxygen atoms, white for carbon atoms and orange for phosphorous atoms.
