# Supplementary material for: Crystal Structure, SAXS and Kinetic Mechanism of Hyperthermophilic ADP-Dependent Glucokinase from Thermococcus litoralis Reveal a Conserved Mechanism for Catalysis
Source: PLoS One. 2013 Jun 20;8(6):e66687. doi: 10.1371/journal.pone.0066687 (PMC3688580; doi:10.1371/journal.pone.0066687)
Supplement: Figure S4 — Nucleotide and sugar binding sites. (DOCX) [file pone.0066687.s004.docx]

**
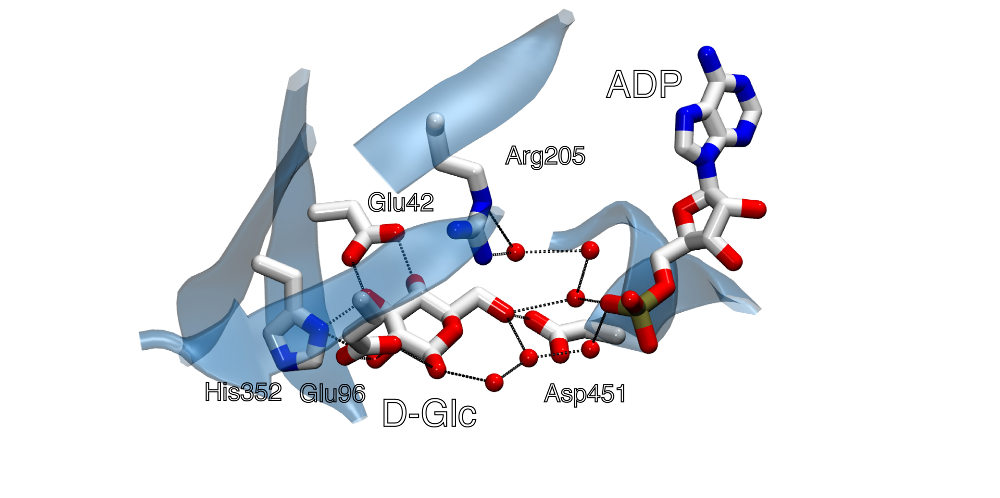
**

**Figure S4. Nucleotide and sugar binding sites in the ternary complex TlGK·Mg·ADP·D-glucose.**  Residue Asp451, the likely catalytic base of the transfer reaction, is only at 2.52 Å from the 6’OH of the sugar. Arg205 can contribute with its positive charge to transition state stabilization, favoring the β-phosphate transfer from the nucleotide to the sugar. Water molecules involved in the reaction are shown as red spheres.
