# Supplementary material for: Crystal Structure, SAXS and Kinetic Mechanism of Hyperthermophilic ADP-Dependent Glucokinase from Thermococcus litoralis Reveal a Conserved Mechanism for Catalysis
Source: PLoS One. 2013 Jun 20;8(6):e66687. doi: 10.1371/journal.pone.0066687 (PMC3688580; doi:10.1371/journal.pone.0066687)
Supplement: Table S1 — Product inhibition parameters. (DOCX) [file pone.0066687.s005.docx]

**Table S1**. **Product inhibition parameters for the TlGK reaction at 40 ºC.**

| **Inhibitor** | **Variable substrate** | **Inhibition** | **K_iq_ (µM)** | **K_ip_ (µM)** |
| --- | --- | --- | --- | --- |
| Mg·AMP | Mg·ADP | C | 28.0 | - |
| Mg·AMP | D-Glucose | NC | 72.2 | - |
| Glucose-6-P | Mg·ADP | NC | - | - |
| Glucose-6-P | D-Glucose | NC | - | 782.3 |

C, competitive inhibition; NC, non-competitive inhibition. K_iq_ and K_ip_ represents the inhibition constants for Mg·AMP and D-glucose-6-P, respectively.
